# Efficacy of combination therapy with pembrolizumab and axitinib for metastatic renal collecting duct cell carcinoma: A report on two cases

**DOI:** 10.1002/iju5.12504

**Published:** 2022-09-26

**Authors:** Shinji Tamada, Daiki Ikarashi, Takashi Tsuyukubo, Kazuhiro Iwasaki, Kazumasa Isurugi, Sadahide Ono, Ryo Takata, Hiromitsu Fujisawa, Wataru Obara

**Affiliations:** ^1^ Department of Urology Iwate Prefectural Central Hospital 4‐1 Morioka‐shi Iwate Japan; ^2^ Department of Urology Iwate Medical University School of Medicine 2‐1‐1 Shiwa‐gun Iwate Japan; ^3^ Department of Pathology Iwate Prefectural Central Hospital 4‐1 Morioka‐shi Iwate Japan

**Keywords:** axitinib, pembrolizumab, renal collecting duct carcinoma

## Abstract

**Introduction:**

Immunotherapy‐based combinations have become the standard first‐line therapy for metastatic renal cell carcinoma. However, combined immunotherapy for renal collecting duct carcinoma had been reported, but its therapeutic efficacy had been unclear.

**Case presentation:**

The first case was a 62‐year‐old man treated with pembrolizumab and axitinib for renal collecting duct carcinoma with multiple bone metastases. After 7 months, the primary and metastatic lesions shrunk and were evaluated as a partial response. The second case was a 71‐year‐old man treated with pembrolizumab and axitinib for renal collecting duct carcinoma with lymph node and lung metastases. After 9 months, the primary and metastatic lesions shrunk and were evaluated as a partial response. In both cases, the tumor cell expression of programmed death ligand‐1 was negative, and CD4^+^ and CD8^+^ cells were observed in the tumor.

**Conclusion:**

Combined immunotherapy with pembrolizumab and axitinib may be effective for metastatic renal collecting duct carcinoma.

**Keynote message**

We presented two cases of partial response to upfront treatment with pembrolizumab and axitinib for metastatic renal collecting duct carcinoma. Combined immunotherapy may be effective for metastatic renal collecting duct carcinoma.

**Abbreviations & Acronyms**
CDC: collecting duct carcinoma
CR: complete response
CT: computed tomography
HE: hematoxylin and eosin
ICI: immune checkpoint inhibitor
IMDC: International Metastatic Renal Cell Carcinoma Database Consortium
irAE: immune‐related adverse event
KPS: Karnofsky performance status
mRCC: metastatic renal cell carcinoma
PD‐L1: programmed death ligand‐1
ORR: objective response rate
TKI: tyrosine kinase inhibitor

## Introduction

The therapeutic strategy for mRCC has expanded in recent years. The combination of immunotherapy and targeted therapy is one of the standard treatments for advanced clear cell RCC.^1^ With regard to the efficacy of pembrolizumab and axitinib, the Keynote 426 trial on mRCC showed an ORR of 60% and a CR rate of 9%.^2^ However, these results were for clear RCC, and information on nonclear RCC was lacking. In particular, the efficacy of pembrolizumab and axitinib for patients with renal CDC remains unclear. Here, we demonstrated continuous response to pembrolizumab and axitinib in two patients with metastatic renal CDC.

## Patient 1

**Fig. 1:**
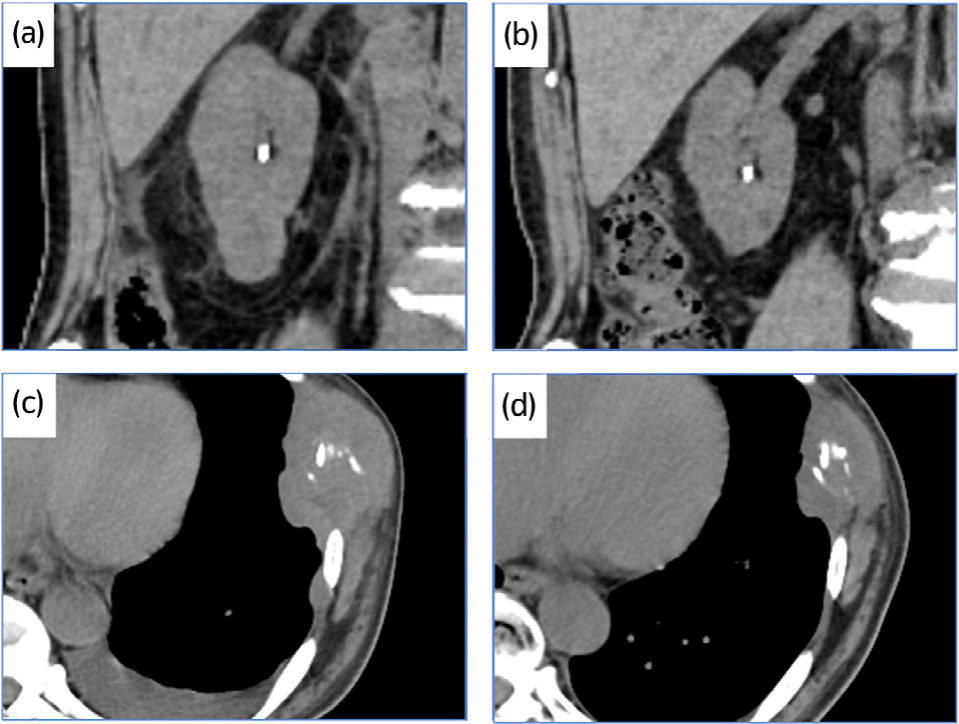
CT images in case 1. There is a right renal mass on initial diagnosis (a) and after 10 cycles of pembrolizumab and axitinib therapy (b). There is a metastatic bone lesion on the seventh rib on initial diagnosis (c) and after 10 cycles (d).

**Fig. 2:**
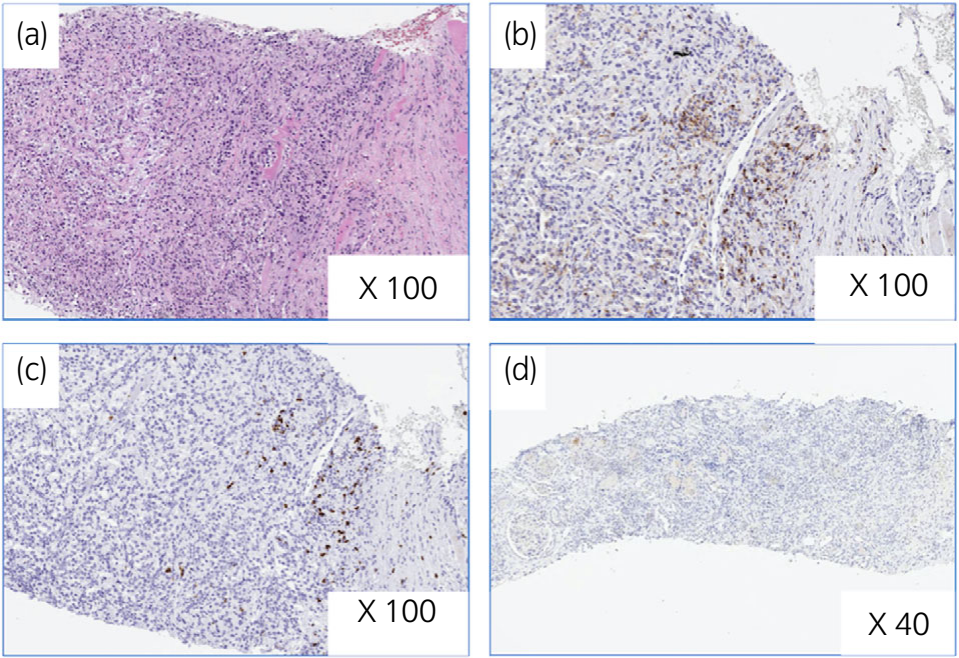
Representative pathological findings of rib mass biopsy in case 1. In case 1, (a) hematoxylin–eosin stain of the pretreatment biopsy specimen shows renal CDC. Immunohistochemical staining demonstrates lymphocyte infiltration of the tumor cells with CD8 (b) and CD4 (c) expressions and the absence of PD‐L1 expression (d).

A 62‐year‐old man presented with left chest pain and appetite loss. He had a history of hypertension. CT scan demonstrated a hypodense right renal mass and multiple rib bone metastases (Fig. 1). CT‐guided biopsy of the rib mass showed a luminal structure that resembled a collecting duct and strong nuclear atypia on HE staining (Fig. 2a), and immunohistochemistry findings that were positive for AE1/AE3 and PAX8 and negative for CD10, and p63; these findings confirmed a diagnosis CDC.

Based on the IMDC prognostic risk grouping, the patient was classified as a poor risk. The patient was started on first‐line combination therapy with pembrolizumab and axitinib, which gradually improved his chest pain and appetite loss. After 10 cycles of pembrolizumab, the renal tumor shrank from 25 to 16 mm, and the rib metastasis shrank from 72 to 53 mm (Fig. 1). The total tumor reduction rate was 30% with achieved partial response. Moreover, the KPS remarkably recovered from 50 to 90. He required administration of levothyroxine because of hypothyroidism, but there were no additional irAEs while on treatment. We performed an additional immunohistochemical examination of the pretreatment tumor sample. The expression of PD‐L1 was negative and tumor‐infiltrating CD4^+^ and CD8^+^ cells were observed in the tumor lesions (Fig. 2b–d). He has continued combined immunotherapy without progression for 7 months.

## Patient 2

**Fig. 3:**
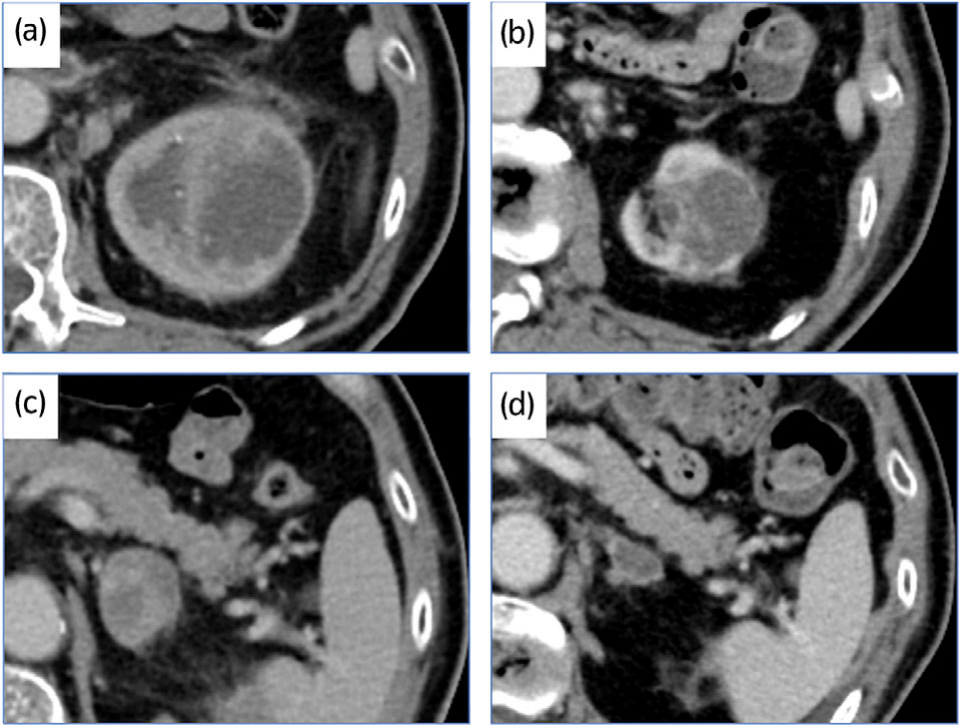
CT images in case 2. There is a left renal mass on initial diagnosis (a) and after 14 cycles of pembrolizumab (b). In addition, there is left adrenal metastasis on initial diagnosis (c) and after 14 cycles (d).

**Fig. 4:**
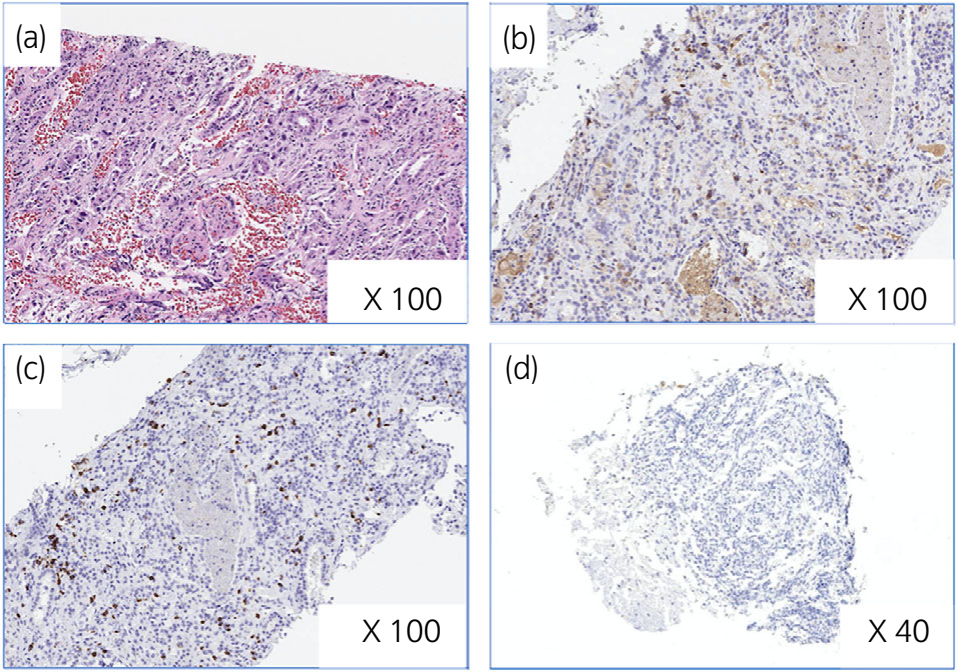
Representative pathological findings of renal mass biopsy in case 2. In case 2, (a) hematoxylin–eosin stain of the pretreatment biopsy specimen shows renal CDC. Immunohistochemical staining demonstrates lymphocyte infiltration of the tumor cells with CD8 (b) and CD4 (c) expressions and the absence of PD‐L1 expression (d).

A 71‐year‐old man presented with back pain. He had a history of hypertension and ureteral stone. CT scan demonstrated a hypodense left renal mass, right adrenal metastasis, and multiple small lung metastases (Fig. 3). CT‐guided biopsy of the renal mass showed a luminal structure that resembled a collecting duct and invasive pattern on HE staining (Fig. 4a), and immunohistochemistry findings confirmed a diagnosis of CDC.

Based on the IMDC prognostic risk grouping, the patient was indicated as a poor risk. The patient was started on first‐line combination therapy with pembrolizumab and axitinib. Because of the poor KPS, axitinib was started at 6 mg/day. After starting combination therapy, he had extreme fatigue and appetite loss. Therefore, we interrupted axitinib. Because his general condition recovered after the interruption of axitinib, we continued only pembrolizumab. After 14 cycles of pembrolizumab, the renal tumor shrank from 70 to 35 mm, the adrenal metastasis shrank from 30 to 20 mm, and the multiple lung metastases resolved (Fig. 3). The total tumor reduction rate was 46% with achieved partial response. He experienced no irAEs while on treatment. The tumor expression of PD‐L1 was negative, and tumor‐infiltrating CD4^+^ and CD8^+^ cells were observed (Fig. 4b–d). He has continued combination therapy without progression for 9 months.

## Discussion

Renal CDC is one of the most aggressive subtypes of renal cancer, with poor prognosis, and over 70% of patients have advanced unresectable stage or metastatic lesions upon initial diagnosis.^3^ Moreover, CDC responds poorly to most chemotherapeutic agents, such as platinum‐based chemotherapy and TKI.^4^, ^5^ Therefore, the development of new treatment options for CDC is needed. Recently, some case reports demonstrated the efficacy of ICI‐based systemic therapy for CDC.^6^, ^7^, ^8^, ^9^ Of note, cases that received first‐line nivolumab and ipilimumab and a second‐line nivolumab treatment were confirmed to achieve CR.^6^, ^7^ In another report, Zhou et al reported a case of metastatic CDC treated with combination therapy of ICI and TKI (i.e., pazopanib and camrelizumab).^10^ These findings showed the potential efficacy of ICI‐based systemic therapy for CDC. For our cases, we selected combination therapy with pembrolizumab and axitinib, which were reported to have high ORR, because the chief complaint was pain secondary to tumor progression. To our knowledge, this was the first case series on metastatic renal CDC that achieved partial response after first‐line combination therapy with pembrolizumab and axitinib.

Renal CDC is known to present as enrichment of the immune signature with infiltration of CD3 and CD8 T cells.^11^ Interestingly, the infiltration percentage of CD3 and CD8 T cells was reported to be higher in metastatic tumors than in nonmetastatic CDCs.^11^ We previously reported the remarkable efficacy of anti‐PD‐1 antibody based on the presence of marked tumor cell infiltration of lymphocytes after treatment.^12^, ^13^ On the contrary, we also reported rapid progression of advanced RCC after treatment with nivolumab following sunitinib; in this case, there were no expressions of PD‐L1 and CD8 on the tumor sample pretreatment.^14^ For the present cases, we found no expression of PD‐L1 on tumor cells, besides CD4 and CD8 lymphocytes infiltration were observed on biopsy specimens; this result was more pronounced in case 2, which consequently responded almost exclusively to pembrolizumab alone. Therefore, the presence of lymphocyte infiltration into the tumor may be associated with the therapeutic efficacy of anti‐PD‐1 antibody therapy. Despite the absence of PD‐L1 expression on tumor cells, our cases showed remarkable clinical responses to combined immunotherapy. However, the correlation between PD‐L1 expression and the response rate to anti‐PD‐1/PD‐L1 antibody has been unclear.^15^ Furthermore, in the updated result of the phase III JAVELIN Renal 101 trial using avelumab and axitinib, an ORR benefit was observed in all patients in the combination arm, regardless of PD‐L1 expression.^16^

We need to mention that the limitation of this case series is that a conclusion cannot be drawn on the response of renal CDC to pembrolizumab and axitinib. In addition, these patients need to be observed in the long term and are yet to undergo nephrectomy. Nevertheless, we believe that the information presented in this report can contribute to further understanding of the potential therapeutic spectrum of combined immunotherapy for renal CDC.

## Author contributions

Shinji Tamada: Conceptualization. Daiki Ikarashi: Conceptualization; supervision. Takashi Tsuyukubo: Investigation. Sadahide Ono: Visualization. Ryo Takata: Supervision. Hiromitsu Fujisawa: Conceptualization; supervision. Wataru Obara: Supervision.

## Conflict of interest

The authors declare no conflict of interest.

## Approval of the research protocol by an Institutional Reviewer Board

Not applicable.

## Informed consent

Informed consent was obtained from the patient for publication of this case report and the accompanying images.

## Registry and the Registration No. of the study/trial

Not applicable.
